# First seizure in elderly patients: Need to treat? Evidence from a retrospective study

**DOI:** 10.1186/s42466-024-00313-8

**Published:** 2024-03-28

**Authors:** Louise Linka, Benedikt Magnus, Nabard Faiz, Lena Habermehl, Panagiota-Eleni Tsalouchidou, Felix Zahnert, Leona Moeller, Kristina Krause, Susanne Knake, Katja Menzler

**Affiliations:** 1 Department of Neurology, Epilepsy Center Hessen, University Hospital Marburg, Philipps-University Marburg, Baldingerstr., 35043 Marburg, Germany; 2 Center for Mind, Brain and Behavior, CMBB, Philipps-University Marburg, Marburg, Germany

**Keywords:** First epileptic seizure, Recurrence, Anti-seizure medication, ILAE, Elderly

## Abstract

**Background:**

The risk of seizure recurrence after a first unprovoked epileptic seizure is reported to be approximately 40%. Little is known about the recurrence risk after a first seizure in elderly patients, who may be at higher risk due to an increased rate of structural lesions, encephalopathy, subcortical arteriosclerotic encephalopathy or brain atrophy.

**Methods:**

In a retrospective approach, the recurrence rate in 304 patients aged 60 years and above who presented with a first seizure between 2004 and 2017 was analyzed. Hierarchical Cox regression was used to investigate the impact of EEG and neuroimaging results, age or the prescription of anti-seizure medication (ASM) on seizure recurrence.

**Results:**

Seizure recurrence rates were 24.5% and 34.4% after one and two years, respectively. Anti-seizure medication was started in 87.8% of patients, in 28.8% despite the absence of clear epileptogenic lesions on neuroimaging or epileptiform potentials in the EEG. Medical treatment significantly reduced the risk of recurrence (hazard ratio = 0.47). Epileptiform potentials in the EEG, epileptogenic lesions in neuroimaging and age had no significant effect on seizure recurrence. Age and the presence of neurodegenerative and psychiatric comorbidities showed a significant association with ASM prescription.

**Conclusions:**

The present data show a strong protective effect of ASM on seizure recurrence in patients above the age of 60, even in the absence of pathologic neuroimaging or EEG results needed for the diagnosis of epilepsy. Treatment with ASM therefore seems beneficial for reducing the recurrence risk in elderly patients. The lack of a significant association between seizure recurrence and epileptogenic lesions might be related to other confounding factors like encephalopathy, subcortical arteriosclerotic encephalopathy, neurodegenerative diseases or brain atrophy.

## Background

Many studies have shown that the lifetime prevalence of an isolated epileptic seizure is about 10% [7]. Recurrence rates after a first seizure are given with 32% after one and 46% after five years in earlier studies [11]. Following the revised definition of epilepsy of the International League Against Epilepsy (ILAE), epilepsy can be diagnosed and treated after a first unprovoked seizure, if the recurrence risk is estimated to be > 60% over the course of 10 years [3, 6, 11]. Usually, presence of epileptogenic lesions in magnetic resonance imaging (MRI) or interictal epileptic discharges (IED) in the electroencephalography (EEG) are considered as the main factors that contribute to a recurrence risk of > 60% and lead to the recommendation of treatment with anti-seizure medication (ASM) after a first seizure [6].

However, most of the studies investigating first seizure and early epilepsy included heterogeneous age groups, without special consideration of older patients. Management of a first seizure in these patients is complicated by increased rates of structural brain changes, brain atrophy, neurodegenerative disorders, comorbidities and potential multiple medication. It remains unclear, if older age itself is associated with an increased chance of a higher seizure recurrence rate, possibly justifying anticonvulsive treatment after a first seizure in this subgroup of patients.

A recent review reports that the prevalence of epilepsy increases with increasing age, reaching a prevalence twice as high as in the adult working population at the age of 75 [12]. Accordingly, the incidence of epilepsy continuously increases above the age of 65 to 180/100,000/year at the age of 85 [12]. After a first seizure, Assis et al. [2] could demonstrate a recurrence rate of 27.5% after 30 days in patients above 60 years of age. Risk factors associated with seizure recurrence in these patients were the number of comorbidities, and the presence of sepsis or psychiatric or cardiac diseases [2]. Little is known about long-term seizure recurrence after a first seizure in elderly patients.

The present study evaluates long-term seizure outcome after a first seizure in patients 60 years or above to identify risk factors associated with seizure recurrence in elderly patients and investigate if immediate anti-seizure treatment, that is often initiated in these patients [15] might be reasonable in this subgroup of patients. These prognostic and therapeutic questions gain importance in the light of the demographic changes and aging populations in many countries [12].

## Methods

### Patients and procedure

The sample comprised patients aged 60 years and above who had presented with a first epileptic seizure at the University Hospital Marburg, Germany. Patients with the diagnosis of an acute symptomatic seizure were excluded. All clinical data were retrospectively collected from the local clinical information system Orbis (Dedalus Healthcare Systems Group, 2021). Clinical data included demographics, type of first seizure (focal, generalized or unknown, provoked vs. unprovoked), the presence of IED identified by EEG, epileptogenic lesions detected by computer tomography (CT) or MRI, type of lesion, ASM and comorbidities and co-medication. Patients who present to our hospital with a first seizure are usually offered a follow-up visit at our epilepsy outpatient clinic after 6–12 months to evaluate recurrent seizures, side effects in case of ASM and driving restrictions. If the patients did not present for a follow-up visit, the patients’ charts in the local documentation software were reviewed including visits unrelated to seizures to extract information on recurrent seizures or seizure freedom that might have been documented there. If recurrent seizures or explicit documentation of seizure freedom could not be retrieved or the patients did not present to our hospital again, the respective patient was labeled as lost to follow-up. The study was approved by the local institutional review board (IRB) and followed the ethical principles of the Declaration of Helsinki. The STROBE guidelines were followed to minimize methodological bias [17].

### Statistical analysis

All statistical analyses were performed using the statistics software SPSS (IBM Corp. Released 2020. IBM SPSS Statistics for Windows, Version 27.0. Armonk, NY: IBM Corp 2020). The significance level was set to α = 0.05. Assumptions for parametric analyses were not violated except for the variable “age”, which was slightly positively skewed. However, given the large sample size, analyses were judged to be robust against this violation [16]. Descriptive and frequency analyses were conducted to examine proportions of clinical features within age groups. Independent samples *t*-tests were run to inspect age differences between patients who were treated with ASM versus those who were not prescribed anti-seizure medication after a first epileptic seizure and to examine possible age differences in patients with versus without a recurrent seizure.

To investigate relationships between clinical and demographic variables across time, we calculated a stepwise Cox regression with recurrence (yes/no) as dependent variable, age at first seizure, EEG and CT/MRI findings in a first block and ASM treatment in a second step (method: enter).

As some patients were treated with ASM after a first seizure, even though there was no epileptogenic lesion in brain imaging and no IED in the EEG, an independent samples *t*-test and chi-square tests of independence were conducted to examine possible associations between medical treatment, clinical characteristics and age of patients upon first seizure in this subgroup. A chi-square test was run to evaluate seizure recurrence in patients with ASM as compared to patients without ASM in the subgroup of patients not fulfilling the diagnostic critera for epilepsy.

## Results

### Clinical characteristics

**Fig. 1:**
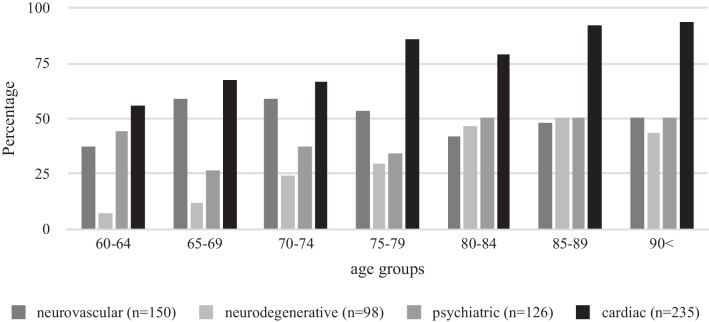
Presence of comorbid diseases across age groups

**Fig. 2:**
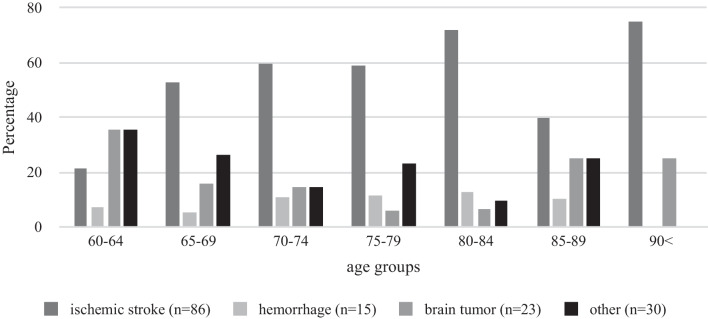
Type of structural lesion identified by MRI/CT across age groups

In total, there were *N* = 328 patients, of which 7.3% diagnosed with an acute symptomatic seizure were excluded from further analyses. The final clinical sample included *N* = 304 patients with a first unprovoked epileptic seizure with a mean age of 77.26 years (*SD* = 8.29, range 60–95; 57.6% female). The mean time between first epileptic seizure and last follow-up was *M* = 43.11 (*SD* = 51.01) months, i.e. 3.2 years. The mean time between first and second seizure was *M* = 17.92 (*SD* = 27.15) months, i.e. 1.1 years. Table 1 presents an overview of clinical characteristics. Figure 1 illustrates the distinct comorbid diseases per age group, showing that cardiac comorbidities were most common across all age groups and that presence of neurodegenerative diseases (e.g., dementia) increased with increasing age. Figure 2 shows the various structural lesions as identified by brain imaging after a first epileptic seizure, indicating that ischemia was the most prominent lesion and increased with increasing age, which is in line with the presence of comorbid neurovascular diseases (Fig. 1).

**Table 1:** Clinical data after first epileptic seizure (total n = 304)

	60–64	65–69	70–74	75–79	80–84	85–90	≥ 90	Total
Recurrence^a^ *N* = 159*	42.9% (9)	16.7% (3)	39.1% (9)	46.2% (18)	37.1% (13)	52.9% (9)	50% (3)	40.3% (64)
Lesion (MRI/CT) *N* = 301	51.9% (14)	55.9% (19)	58.7% (27)	53.1% (34)	49.3% (33)	42.6% (20)	50% (8)	51.5% (155)
IED in EEG *N* = 285	18.2% (4)	26.5% (9)	42.2% (19)	35% (21)	34.4% (21)	38.3% (18)	31.3% (5)	34% (97)
ASM treatment *N* = 304	81.5% (22)	82.4% (28)	93.6% (44)	85.9% (55)	85.3% (58)	91.7% (44)	100% (16)	87.8% (267)

Number of patients with respective characteristics appear in brackets behind percentages

MRI, magnetic resonance imaging; CT, computer tomography; IED, interictal epileptic discharges. EEG, electroencephalography. ASM, anti-seizure medication

^a^At latest documented follow-up

*Total N of patients where characteristic is/is not documented.

### Recurrence

**Fig. 3:**
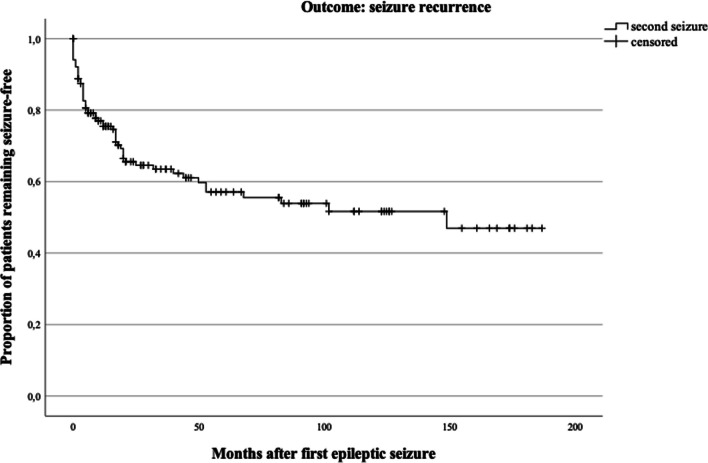
Kaplan–Meier curve on recurrence rate after first epileptic seizure (months)

Within the clinical sample, 40.3% (*n* = 64) of all patients had a documented recurrent epileptic seizure at latest follow-up. The Kaplan–Meier curve (Fig. 3) illustrates that within the first year, 24.5% of patients experienced a recurrent seizure and after two years, 34.4% had a documented recurrent seizure (total *n* = 153).

**Fig. 4:**
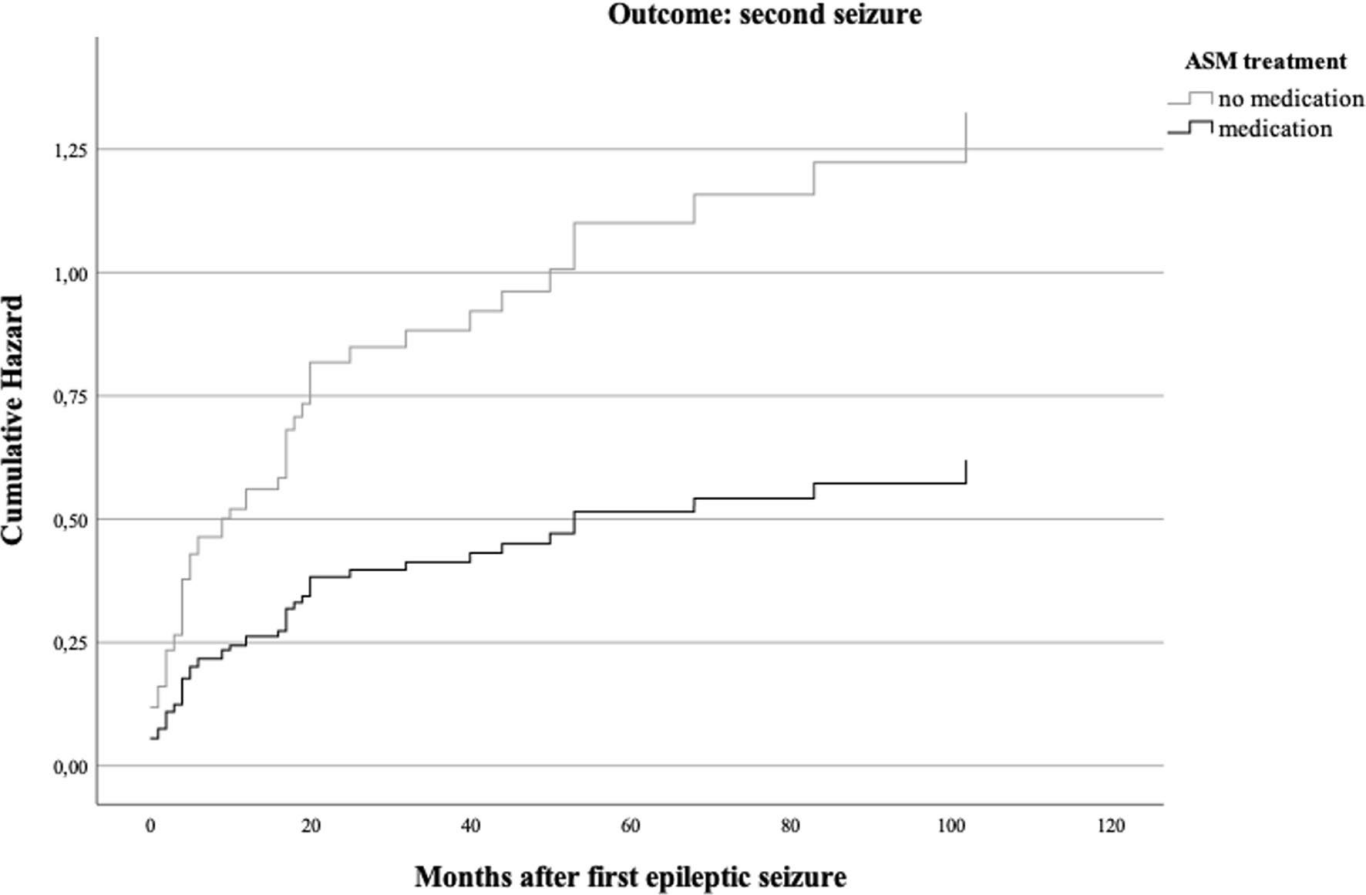
Hazard ratios for seizure recurrence depending on ASM treatment

An independent-samples *t-*test showed that patients experiencing a recurrent seizure were not significantly older or younger than patients who remained seizure-free (*t*(157) = − 0.97, *p* = 0.332), equal variances assumed. A stepwise Cox regression model showed that the first block including epileptogenic lesions on CT or MRI scans, IED in EEG and age of patients did not significantly contribute to seizure recurrence across time (χ^2^(3, *n* = 144) = 1.80, *p* = 0.615). Entering ASM in the consecutive block showed a statistical trend (χ^2^(1, *n* = 149) = 3.74, *p* = 0.053). Within the whole model, ASM treatment significantly contributed to predicting seizure recurrence (*z*(1) = 4.18, *p* = 0.041, hazard ratio = 0.47, 95% CI = 0.23–0.97; see Table 2). Figure 4 illustrates hazard ratios for seizure recurrence depending on ASM treatment across time.

**Table 2:** Cox regression (full model), dependent variable: second seizure

Covariates	HR	95% CI	Wald	*p*-value
Lesion in MRI/CT	1.29	0.72–2.33	0.72	0.40
IED in EEG	1.51	0.85–2.69	1.93	0.16
Age	1.02	0.98–1.05	0.79	0.37
Use of ASM	0.47	0.23–0.97	4.18	0.041*

HR, hazard ratio; CI, confidence interval; MRI, magnet-resonance imaging; CT, computer tomography; IED, interictal epileptiform discharges; EEG, electroencephalogram; ASM, anti-seizure medication

*Significant at α = 0.05

### Treatment with ASM

**Fig. 5:**
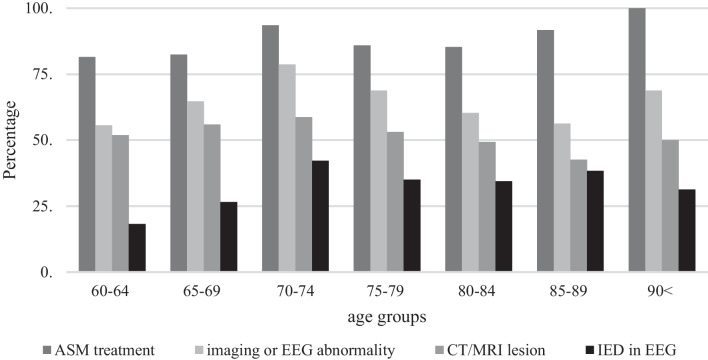
Diagnostic features and ASM treatment across age groups

Of all elderly patients with a first epileptic seizure, 87.8% (*n* = 267) were treated with anti-seizure medication. Of these, 71.2% showed either an epileptogenic lesion in CT/MRI or IED in EEG. There was no significant difference in age between patients treated with ASM and those who did not receive ASM after a first seizure (*t*(157) = − 0.85, *p* = 0.40, equal variances assumed). Figure 5 illustrates the diagnostic features and ASM treatment across age groups within the elderly.

Of all patients treated with ASM, 77 (28.8%) did not have lesions identified by brain imaging or IED in the EEG. Treatment was therefore not in line with the current recommendation in these patients as the diagnosis of epilepsy usually requires two unprovoked seizures or one seizure and the presence of epileptogenic lesions in brain imaging or IED in the EEG. The relative amount of patients receiving ASM in the absence of EEG or imaging abnormalities ranged from 16% (70–74 years) to 41% (85–89 years). In this subgroup of patients, 67.5% (*n* = 27) remained seizure free and 32.5% (*n* = 13) reported seizure recurrence, while *n* = 37 were lost to follow-up. There was a statistical trend towards a reduced recurrence rate as compared to the 17 patients without epileptogenic lesions or IED who did not receive ASM and in whom follow-up information was available (recurrence rate 32.5% with ASM vs. 58.8% without ASM, χ^2^ (1, *n* = 57) = 3.44, *p* = 0.064).

Inspecting the patient group without imaging lesions and/or IED in EEG more closely, an independent samples *t*-test revealed that those patients receiving ASM were significantly older (*M* = 78.71, *SD* = 8.48) than patients who were not treated with ASM (*M* = 74.73, *SD* = 8.87), *t*(105) = − 2.15, *p* = 0.034, equal variances assumed. Further chi-square tests of independence with Yate’s Continuity Correction showed that there was a significant association between ASM prescription and neurodegenerative comorbidities (χ^2^ (1, *n* = 107) = 6.46, *p* = 0.011, *phi* = 0.27) and also a significant association between ASM prescription and psychiatric comorbidities (χ^2^ (1, *n* = 107) = 9.07, *p* = 0.003, *phi* = 0.31). Of those patients with a neurodegenerative disease, 87.5% received ASM as opposed to 62.7% of patients without a neurodegenerative disease. Similarly, 87.5% of patients with a psychiatric comorbidity received ASM, while this was only the case for 59.3% of patients without a psychiatric comorbidity. There were neither significant associations between ASM prescription and neurovascular comorbidities (*p* = 0.196) nor between ASM prescription and cardiac comorbidities (*p* = 0.183) following chi-square tests of independence with Yate’s Continuity Correction.

## Discussion

The present study shows seizure recurrence rates of 24.5% and 34.4% after one and two years in patients aged 60 years and above with a first unprovoked seizure. In this patient group, 87.8% were treated with ASM after the first seizure, 28.8% of those despite the absence of clear epileptogenic lesions in neuroimaging and the absence of epileptiform potentials in the EEG. However, medical treatment had a protective effect on seizure recurrence, reducing the recurrence risk with a hazard ratio of 0.47. Age was not significantly associated with the recurrence rate. Also, epileptogenic lesions in neuroimaging and epileptiform potentials in the EEG did not have a significant effect on seizure recurrence.

### Recurrence rates in patients above the age of 60

Recurrence rates after a first unprovoked seizure have been analyzed in several studies [1, 8, 9, 11]. A population-based study suggested a recurrence rate of 36–37% at one year and 43–45% at two years after a first unprovoked seizure [9]. Krumholz et al. [11] could show that recurrence rates after a first seizure were 32% after one and 46% after five years including both treated and untreated patients [11]. However, these studies mainly included patients who received older ASM and were based on the old definition of epilepsy, which required at least two unprovoked seizures. Also, these studies included all age-groups, resulting in a younger, heterogeneous patient group [1, 8, 9, 11]. Results might therefore not be transferable to older patients as epilepsy is more common above the age of 65, reaching a prevalence twice as high as in the adult working population at the age of 75 [12]. Also, the incidence of epilepsy starts to increase at the age of 65 and reaches its maximum of 180/100,000/year at the age of 85 [12]. These numbers imply a higher recurrence risk after a first seizure in these patients. Also, the increasing prevalence of structural brain lesions like ischemic stroke or neurodegenerative disorders might influence the recurrence rates [12]. Moreover, older patients more often live alone and seizure symptoms are often more subtle in older patients and might therefore not be noticed by the patient or relatives, leading to an underestimation of recurrence rates [14].

After a first seizure, Hart et al. [8] could show that the risk of seizure recurrence is influenced by age, the risk being highest in patients below 16 or above 59 years [8]. Investigating the short-term recurrence risk, Assis et al. [2] could demonstrate a recurrence rate of 27.5% after 30 days in patients above 60 years of age. After 10 years, recurrence rates of 37.4% are reported in elderly patients [10]. It remains unclear if these older data can easily be applied to current patients, as the definition of epilepsy [6] has changed in the meantime, allowing for the diagnosis and treatment of epilepsy after a first seizure depending on EEG and imaging results. This earlier treatment along with the introduction of new ASM might have influenced the recurrence rate. In our study, recurrence rates were relatively low with 24.5% after one and 34.4% after two years. This low recurrence rate is possibly related to the high number of patients receiving ASM right after a first seizure and the strong influence of medical treatment on preventing seizure recurrence.

### Factors associated with seizure recurrence

Usually, seizure recurrence is thought to be influenced by the presence of an epileptogenic lesion in neuroimaging or the detection of IED in the EEG. Either of these findings justifies treatment with ASM immediately after a first, unprovoked seizure following the revised definition of epilepsy of the ILAE [6]. Neither the effect of neuroimaging results nor of IED on the recurrence rates could be confirmed in the present study. The lack of an association between seizure recurrence and structural brain lesions in our patients might be related to the high incidence of ischemic stroke, as this etiology was associated with the highest percentage of long-term seizure freedom in patients with focal epilepsies of different etiologies [5]. Future research might focus on the distinct contributions of specific lesions to seizure recurrence more in depth.

The influence of MRI and EEG findings in older patients might also be confounded by the increased number of structural brain lesions, chronic vascular disease, brain atrophy, neurodegenerative diseases or multi-morbidity. In older patients, short-term seizure recurrence was also reported to be associated with number of comorbidities, sepsis, psychiatric or cardiac diseases [2]. Possibly, the impact of neuroimaging or EEG findings on seizure recurrence is overestimated in this patient subgroup and should not exclusively guide the decision to initiate treatment with an ASM.

### Anti-seizure medication

We could show that medical treatment significantly reduced the recurrence risk in older patients after a first seizure. This strong effect justifies the initiation of an ASM after a first seizure in this age group. The strong effect of ASM might in part be related to the high incidence of ischemic stroke, which was reported to be associated with a good response to ASM treatment [5, 18]. Even though the favorable effect of ASM for the treatment of post-stroke epilepsy as compared to other focal epilepsies has recently been questioned [18], our data support this favorable effect in elderly patients with a high percentage of post-stroke epilepsies. Future research is warranted to further investigate the outcome and predictors of post-stroke epilepsies [18].

Earlier evidence shows that older patients are often prescribed ASM after a first seizure, even though evidence supporting or challenging this clinical decision is lacking [15]. In line with this observation, 87.8% of patients were treated with anticonvulsive medication in the present study, 28.8% of those in the absence of clear epileptogenic lesions on neuroimaging or IED on the EEG. This clinical decision was related to the presence of neurodegenerative or psychiatric diseases and might have been guided by the observation of increased short-term seizure recurrence in patients with comorbidities, sepsis, psychiatric or cardiac diseases reported earlier [2]. The current german guidelines do not generally recommend an ASM after a first seizure not fulfilling the criteria for epilepsy even in the elderly, but discuss that treatment might be considered in cases with extensive vascular leukencephalopathy [13]. In the subgroup of patients not fulfilling the diagnostic criteria for epilepsy, our data showed a trend towards reduced recurrence rates in patients who received an ASM, which did not reach the significance level, possibly due to very small sample sizes. Future studies should therefore focus on this subgroup of patients to evaluate positive and negative effects of treatment in the absence of clear epileptogenic lesions and IED in MRI and EEG.

Future studies should also include the burden of side effects of ASM in this patient group like dizziness, the impact on cognition or vigilance or laboratory results. Also, the interaction of ASM with co-medication and a possible aggravation of symptoms related to comorbidities needs to be taken into account.

### Conclusions and limitations

The present study is a retrospective study. As retrospective studies usually show higher recurrence rates than prospective studies, possibly due to the higher proportion of seizure-free patients lost to follow-up as compared to patients with seizure recurrence [4], the present study might overestimate the seizure recurrence rate. On the other hand, seizure recurrence rates might be underestimated due to the more subtle seizure semiology in older patients and more common social isolation [12, 14]. Prospective studies evaluating seizure recurrence in elderly patients are therefore warranted.

The present study investigates older patients exclusively after a first epileptic seizure in order to evaluate recurrence rates, factors influencing seizure recurrence and the effect of ASM in this growing patient group. Compared to older studies and studies including different age groups [9, 11], recurrence rates were relatively low with 24.5% after one and 34.4% after two years. We could show a strong protective effect of ASM treatment in these patients, justifying early treatment after a first unprovoked seizure.

## Data Availability

The datasets used and/or analysed during the current study are available from the corresponding author on reasonable request.
